# Biosensor-Assisted Method for Abdominal Syndrome Classification Using Machine Learning Algorithm

**DOI:** 10.1155/2022/4454226

**Published:** 2022-01-28

**Authors:** Charu Gandhi, Sayed Sayeed Ahmad, Abolfazl Mehbodniya, Julian L. Webber, S. Hemalatha, Haitham Elwahsh, Basant Tiwari

**Affiliations:** ^1^Department of CSE & IT, Jaypee Institute of Information Technology, Noida, India; ^2^College of Engineering and Computing, Al Ghurair University, Dubai, UAE; ^3^Department of Electronics and Communication Engineering, Kuwait College of Science and Technology (KCST), Kuwait, Kuwait; ^4^Graduate School of Engineering Science, Osaka University, Osaka, Japan; ^5^Department of Computer Science and Engineering, Panimalar Institute of Technology, Chennai, Tamil Nadu, India; ^6^Faculty of Computers and Information, Kafrelsheikh University, Kafrelsheikh, Egypt; ^7^Department of Computer Science, Hawassa University, Awasa, Ethiopia

## Abstract

The digestive system is one of the essential systems in human physiology where the stomach has a significant part to play with its accessories like the esophagus, duodenum, small intestines, and large intestinal tract. Many individuals across the globe suffer from gastric dysrhythmia in combination with dyspepsia (improper digestion), unexplained nausea (feeling), vomiting, abdominal discomfort, ulcer of the stomach, and gastroesophageal reflux illnesses. Some of the techniques used to identify anomalies include clinical analysis, endoscopy, electrogastrogram, and imaging. Electrogastrogram is the registration of electrical impulses that pass through the stomach muscles and regulate the contraction of the muscle. The electrode senses the electrical impulses from the stomach muscles, and the electrogastrogram is recorded. A computer analyzes the captured electrogastrogram (EGG) signals. The usual electric rhythm produces an enhanced current in the typical stomach muscle after a meal. Postmeal electrical rhythm is abnormal in those with stomach muscles or nerve anomalies. This study considers EGG of ordinary individuals, bradycardia, dyspepsia, nausea, tachycardia, ulcer, and vomiting for analysis. Data are collected in collaboration with the doctor for preprandial and postprandial conditions for people with diseases and everyday individuals. In CWT with a genetic algorithm, db4 is utilized to obtain an EGG signal wave pattern in a 3D plot using MATLAB. The figure shows that the existence of the peak reflects the EGG signal cycle. The number of present peaks categorizes EGG. Adaptive Resonance Classifier Network (ARCN) is utilized to identify EGG signals as normal or abnormal subjects, depending on the parameter of alertness (*μ*). This study may be used as a medical tool to diagnose digestive system problems before proposing invasive treatments. Accuracy of the proposed work comes up with 95.45%, and sensitivity and specificity range is added as 92.45% and 87.12%.

## 1. Introduction

Human physiology comprises the nervous system, cardiovascular system, respiratory system, and digestive system. The digestive system, among these systems, is one of the most powerful systems where the stomach plays a vital part with its accessories such as the esophagus, duodenum, small intestines, and large intestines. The digestive system consists of the gastrointestinal tract, the mouth twisting pipe to the anus, and other organizations that assist the body to break down and absorb food. Food and drinks must be transformed into smaller molecules of nutrients before they can be absorbed and transported to cells throughout the body for food. Digestion is when food and beverage are divided into smaller pieces to allow the body to utilize them to construct and feed the cells and provide energy. However, worldwide, many individuals have stomach illnesses linked with gastric motility abnormalities, such as dyspepsia (improper digestion), inexplicable nausea (sensation failure), vomiting, abdominal pain, stomach ulcer, and gastroesophageal reflux disorders. Some of the techniques used to identify anomalies include clinical analysis, endoscopy, electrogastrogram, and imaging. Of the abovementioned techniques, electrogastrogram (EGG) is noninvasive and cost-effective [1]. Electrogastrogram is the registration of electrical impulses that pass through the stomach muscles and regulate the contraction of the muscle. The electrode senses electrical impulses from the stomach muscles, and the EGG is recorded to investigate digestive system problems. The research is carried out with diseases such as bradygastria, dyspepsia, nausea, tachygastria, ulcers, and vomiting. EGG data are evaluated using statistical parameters, method of wavelet transformation, and approach to the neural network.

### 1.1. Digestive System

**Figure 1:**
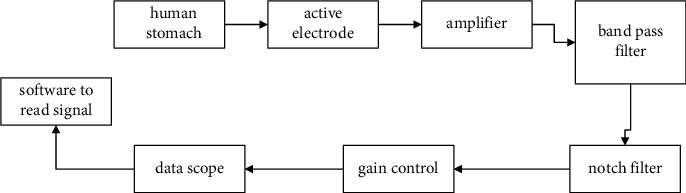
EGG recording procedure.

The human body has to survive with food. However, the food in the body is just a tiny component of the process. The meal must be divided into chemical substances that the body can utilize. This entire process is known as digestion. As shown in Figure 1, the digestive system comprises several organ breakdowns and disposal of chemical ingredients of meals, including stomach, pancreas, liver, gallbladder, small gut, and considerable intestinal fluctuation. Digestion in the mouth starts. The chewed meal is lubricated and humidified by saliva, a watery mucus, and enzyme composition. The second phase of digestion takes place in the stomach, in which stomach fluids are separated and combined with the meal for fluid termed chyme. It moves from the stomach into the duodenum, where the liver and pancreas handle more enzymes. The liver creates bile to break down fats stored in the gallbladder and goes into the duodenum via the bile duct. The pancreas generates and releases enzymes for the digestion of proteins and carbohydrates. After treating the pancreatic enzymes and bile, chyme passes via the small intestine. The small intestine is treated with some extra enzymes produced by the intestinal wall, and the digestive process is over. Absorption occurs in the small gut. In the large intestine, water is eliminated. Digestion performs an essential function in the stomach. It appears like a flattened ball when it is empty, but when filled, it can contain approximately two-quarters of food and drink for 1 foot and 6-inch width. The stomach comprises chemical and mechanical activity. Several substances in the stomach, notably pepsin, rennin, and lipase digestive enzymes, combine to break up food. In addition, hydrochloric acid provides an appropriate environment for the enzymes and supports digestion. The watery mucus protects the muscle walls of the stomach from the digestion of the acid or enzymes. The mechanical activity of the stomach muscles constraints and relaxes in a continual mixture, whipping and churning the stomach's substance in the chyme.

The EGG is comparable to the cardiovascular electrocardiogram (ECG). It captures electric impulses that pass through the stomach muscles that regulate the muscle's contractions. It also monitors the activity of the stomach wall before and after the intake of meals. EGG traces have a 3-cycle frequency of sinusoidal waveforms per minute. Clinical investigations have demonstrated a good relationship between these cutaneous recordings and those obtained by serially implanted electrodes. EGG is assessed as a regular electric rhythm produced by stomach muscles in ordinary people, and the strength (voltage) of the electric current rises after food, and in patients with stomach muscle disorders, the rhythm is erratic, or electric power increases after a meal [2, 3]. There are no adverse effects of EGG recording, and the research is painless.

EGG is presently used in research and clinical settings since it is an efficient technique for stomach electrophysiology and gastric motility disorders pathophysiological studies [4]. Since the first EGG recording development was particularly sluggish in this area compared with other electrophysiological cutaneous measures because of its difficulties in acquiring data, there is a lack of knowledge of EGG at unique frequency and amplitude. Parameters are being discussed, and EGG's clinical use is still being studied [5].

### 1.2. The Motivation of the Proposed Work

Therefore, a frequency range must be established for ordinary individuals and dysrhythmias, EGG recording standardization, and sophisticated analytical techniques for extracting and interpreting quantitative EGG data. Additional EGG result studies are also required to establish the use of EGG in the therapeutic context. Today, doctors and biological researchers are interested in the quantitative analysis of EGG. Currently, electrogastrogram application in India is not performed up to date since it is a completely noninvasive method to study digestive problems. Many researchers follow this subject to get accurate findings. Acquisition and analysis of EGG to help the physician diagnose digestive system problems at an early stage with considerable accuracy.

## 2. Background Analysis

The author's limb records the EGG of a five-week-old kid with pyloric stenosis. The EGG seemed comparable to an electrocardiogram (ECG) with progressively shifting baselines. Smout et al. demonstrated that when contractions occur, the amplitude of the EGG rises [6]. Chen et al. have developed a new method of spectrum analysis based on an adaptive, moving average model [7]. This technique gave better precision and more accurate information regarding frequency changes in electric stomach activity. It is beneficial for identifying short-term dysrhythmic occurrences of stomach activity.

### 2.1. Analysis of Electrogastrogram

The first to use the spectrum analysis method to EGG was Stevens and Worrall and then to analyze EGG data using Fourier transform [8]. Chen et al. conducted arithmetical analyses to examine the change in EGG designs between individual subject collections [9]. *T*-tests were performed for students in pairs and unpaired to evaluate differences and assign statistical significance. Hrair Simonian et al. (2004) stated the definition of average values as mean ± 2 [10]. The standard abnormal deviation is calculated when one of the values above is beyond the range. Ding et al. employed an electrogastrography to detect slow stomach waves, and the authors developed a multiresolution technique to deconstruct the EGG signal using the Daubechies wavelet function [11]. Zhenghu has created a novel wavelet-based treatment technique of EGG signals with an excellent application viewpoint. It is easy and quick to produce accurate charts and frequency identification features for refining [12]. Kania et al. have investigated the significance of the proper selection of mom's wavelet for decomposing the ECG signal noise [13]. The researchers concluded they got a high-quality signal on the first and fourth degradation levels for the wavelet db1 and sym3 for the fourth degradation level.

Zhongjia et al. utilized the Social Sciences Statistical (SPSS) package for the examination of alteration (ANOVA) and range analysis to show in what way various pressure values and moisture content affect the forming density and to assess the significance of the two variables to shape biofuel density [14]. Xing-ce et al. conducted a brain vascular segmentation preprocessing technique based on the parametric statistical model [15]. After the preprocessing stage, the writers examined the brain picture as an input to the parameterized statistical model and secreted the tiny branches of their brain vessels.

Elsayad used neural Learning Vector Quantization (LVQ) networks to identify ECG dataset arrhythmias [16]. For a future study on any biosignals, the experimental findings suggest LVQ algorithms. Ramanathan et al. have categorized the lung sound using the ANN architecture wavelet coefficients [17]. For the standard or pathological categorization of EGG signals, Tsung-Nan Lin et al. observed the optimum outcome by having six to seven concealed neurons in the network [18]. Slow convergence and key user-dependent factors are barriers to distinguishing normal and aberrant signals. Dutta et al. utilized a heartbeat diagnostic medical diagnostic tool to accomplish an accurate and timely diagnosis of heart arrhythmia to provide a patient who uses an extractor combined with an artificial neural network (ANN) classification with adequate medical care [19]. Based on a sliding dot product method, the attribute filter uses the frequency-domain data of cross-spectrum. Ahsan et al. presented the method of identifying various hands for complicated EMG-based classification and pattern recognition applications with the aid of an artificial neural network (ANN) [20]. The authors utilized BPN with the training method Levenberg-Marquardt for gesture identification. Shiau et al. conducted a cardiac gesture examination for all serial and patient pictures utilizing the BPN network analysis [21]. Barrea and Barrea utilized a local fuzzy c-means clustering to analyze the spectroscopic data to verify a novel medication for prostate malignancy called clioquinol (CQ) [22]. To address the drawbacks of this current work, the work suggested an experimental set-up for recording electrogastrogram cutaneously with patients and studying digestive systems diseases to see the deviation of stomach signal from average to abnormal. To build an EGG database containing bradygastria diseases, dyspepsia, nausea, tachygastria, ulcer, and vomiting disorders along with the normal subject, Naive Bayesian Classification to conduct statistical analysis and categorize the EGG signals based on higher-order momentum (NBC), and Continuous Wavelet Transform (CWT) applications to EGG signals for disorder identification and categorization, they proposed the appropriate neural network and training method using an unsupervised neural network of adaptive resonance theory (ART-NN) (LVQ) and analyze the performance of BPNN for the nine training algorithms, i.e., gradient descent with momentum and adjustable learning rate, backing sprouting (GDX), resilient back spraying (RP), conjugate backpropagation gradient with updates from Fletcher-Reeves (GCF), a combination of backpropagation gradient with updates from Polak-Ribiere (CGP), the connection of backpropagation gradient with restarts from Powell-Beale (CGB), and scaled juxtaposition to use the clustering method fuzzy c-means to discrimination between standard and arrhythmic EGG signals to enhance classification efficiency.  Table 1 shows the various comparison rate of the existing methodology.

The network was trained on two classes of EGG signals and used to predict 102 samples in the patients. An average EGG signal (normogastria) was identified as one of two signal classes in all of the investigations described above, with the aberrant EGG signal designated as the other. For this study, we will treat the states of normogastria, bradygastria, and tachygastria independently to evaluate two- and three-class problems. The electrogastrography signals are divided into three groups for three-class signal classification and two groups for two-class signal classification, respectively (normal and abnormal). We successfully classify two-class and three-class EGG signals using statistical machine learning algorithms such as Logistic Regression, SVM, and KNN. We compare their performances to suggest which applied ML algorithm is the most effective for a multiclass EGG signal classification. The total accuracy, F1 score, precision, and recall are the performance metrics taken into consideration in this research.

## 3. Proposed Work

Most individuals throughout the globe suffer health issues, mainly owing to dietary intake and digestive system diseases. Today, the Endoscope technique is used to study the issues of a laborious, costly, and intrusive approach to digestive system disease. To detect the electric signal cutaneously from the stomach, a noninvasive, inexpensive, and painless electrogastrogram (EGG) method has been developed as an initial method to investigate gastric disorders before encouraging the Endoscope procedure for noninvasive gastric disease and benign tumors. Because of its noninvasive nature and the recent improvements in EGG recording and computerized analysis methods, EGG has become a valuable researcher's tool for studying the electrophysiology of gastric motility disorders and is now used in both research and therapeutic settings. Contrary to ECG, EEG, and EMG, electrogastrogram signal in any database is not accessible. This study aims to develop a technique for obtaining high-quality EGG noise-free signal, cheap cost, and reduced complexity of purchases for any individual with or without symptoms for preliminary examination of digestive system problems before invasive processes.

The stomach may be monitored closely, intraluminally, or cutaneously by gastric myoelectric activity (GMA). The serosal record may be achieved by surgically inserting electrodes on the serosal surface of the stomach. The intraluminal record may be obtained by incubating a catheter with electrodes. Suction is typically used to provide good contact between the electrodes and the mucosal wall of the stomach. The serosal and intraluminal elements may record slow waves and spikes because they constitute a limited number of smooth muscle cells with myoelectric activity. These techniques are intrusive, and their applicability in animals and laboratory environments is restricted. EGG, a cutaneous measurement of GMA by surface electrodes, is extensively utilized in people and clinical settings since it is noninvasive and does not disrupt continuous stomach action [23]. Various validation studies have shown the accuracy of the EGG by comparing it to the mucosal and serosal electrode record [24]. Reproducibility of the EGG recording, without substantial daily fluctuations, was shown. In adults, gender and age seem to have little effect on the EGG. The recording is done using surface electrodes noninvasively.

One of the primary goals was to categorize electrogastrography signals with high accuracy under both a two-class and a three-class classification model, which was accomplished. The electrogastrography signals are divided into three groups for three-class signal classification (normogastia, bradygastria, and tachygastria) and two groups for two-class signal classification (normogastia, bradygastria, and tachygastraria) (normal and abnormal). Our machine learning techniques for classifying two and three classes of EGG signals included Logistic Regression, SVM, and KNN, which were all implemented. As a consequence of our experiments, we find that the SVM method is the most accurate in classifying the two- and three-class signals, with accuracy rates of 100 percent and 92.11 percent, respectively. The SVM algorithm attained a maximum F1 score, precision, and recall value of 100 percent and 92 percent, respectively, for the two and three classes of EGG signals, using the SVM technique. In addition, SVM effectively identified EGG signals with a high degree of separability of 100 percent and 92 percent in the two-class and three-class classifications, respectively, with a high degree of separability of 100 percent and 92 percent. Following our investigation, we have concluded that SVM can be effectively used to reliably classify multiclass EGG signals and forecast the signal features of such signals.

### 3.1. Materials and Methods

#### 3.1.1. Image Acquisition

By the Helsinki declaration [25], EGG data acquisition shall be carried out by explaining the procedure of all subjects (normal and abnormal) under the guidance and monitoring of renowned hospital gastroenterologists who have been admitted to participate in the study. More than a thousand sample EGGs, including patients and ordinary individuals from both male and female categories of various age groups, were included in this recording, as indicated in Table 2 [26–28]. Electrogogram is recorded with a minimum length of half an hour under preprandial (before meal) and postprandial (after food) conditions.

Figure 1 shows the electrogastrogram recording set-up utilizing Method E. Active electrodes hit the abdominal bioindication through motility on the skin. The electrodes' output consists of input into the SCU, an instrument amplifier, a passband filter, a band-reject filter, and gain control. SCU contains the process such as adaptation to the range, filtering, conversion, amplification, insulation, and other operations required for subsequent sensors processing. A device amplifier is employed in SCU for improving the electrode sensing potential. With the aid of a voltage signal as input, an amplifier produces a signal of linear variant at the output. It is a PLL amplifier, typically with high input impedance, short drift, and increased frequency rejection. A *filter* is an instrument that transmits frequencies in a definite range and limits frequencies beyond this scope. A band-reject filter is also termed a Notch filter employed to remove a specific frequency segment of a signal. Also, it can be employed in minimizing or avoiding feedback. In many electrical devices, the control of gain is an adaptive mechanism that averages the signal level at the output, thereby adjusting the gain to a suitable level for different input signal levels, conditioning the signals almost for all data with normalized sensor signals filtered to appropriate levels compatible with digital-analog conversions into computer processor recording and analysis.

Approximately 1000 individuals were examined based on the gastroenterologist's previous knowledge. The EGG database comprises about 500 individuals, with an average of 70 per category of 6 diseases and a normal category based on the conditional probability based on Baye's theory. The Sri Ramakrishna Hospital was guided to capture EGG signals at the Department of Biomedical Engineering, Sri Ramakrishna Engineering College, Coimbatore. Initially, the sample collected from MEDINDIA Hospital and PSG Hospital, Coimbatore, generated the database. The average and pathological thresholds are established following the physician from the database. This was utilized as the benchmark or the fundamental truth. The recording stability has been confirmed in PSG Hospitals and MEDINDIA Hospitals, Coimbatore. 60 samples per minute of EGG data were utilized for normal and disorder patients as classification inputs.

#### 3.1.2. Feature Extraction with Optimization

Continuous Wavelet Transformation (CWT) has been created as an alternate FT method to decrease the complexity of data extraction. The word wavelet refers to a bit of wave. The smallness refers to the condition of a finite length of this function. The wave refers to the state of oscillation. A feature (wavelet) multiplies the signal to obtain the CWT of a signal, and the transform is calculated independently for various parts of the time domain signal.

Four subbands result in a single wavelet decomposition of ROI. Daubechies wavelet filter [29] is used for decomposition in order 2. The subband, which displays the changes with the most apparent appearance between various textures, has the most significant difference in the histogram. This subband is selected to be further processed.

Haralick et al.'s method to spatial dependence matrix in grey color (1973) is a well-known statistical technique for collecting secondary texture data from images used for this study [30]. The SGLDM comprises the assessment of the discrete second-order function.

**Figure 2:**
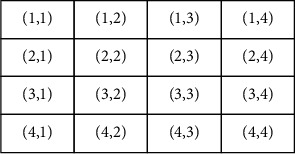
Horizontal value of the input signal.

The collection of all horizontally adjacent resolution cells separated by distance is shown in Figure 2. Figure 2(a) displays a grey image of four to four of 0 to 3 tones. Figure 2(b) shows a grey-tone spatial dependence matrix (b). For example, at (2, 1) distance 1 of the horizontal PH matrix, the total number of times two grey color tones, with values of 2 and 1, occurred horizontally adjacent to each other. We count the number of cell resolution pairs in RH for calculating this number, such that the first resolution of the pair has a grey ton 2 and the second resolution of the pair's cell is grey ton 1. Figures 2(c)–2(f) show spatial addiction matrices of all four distances of one grey tone.

The coincidence conditions are determined for offset angles of 0 ∘ to 135 ∘ with a step value of 45 ∘ for a certain offset distance. 14 SGLDM measurements are generated from each of these matrices. Each measure is characterized by the mean and the range (difference between maximum and minimum) across four offset angles; this results in 28 characteristics for, respectively, offset distance (Table 3).

Features for offset distances of 1, 2, 3, and 4 are computed. This gives a total of 112 characteristics. The second corner feature reflects the consistency of the textures. Contrast quantifies the number of local differences in a picture. Correlation is a grey-tone linear picture dependency metric. Variance is a measure of distribution dispersion. The contrary difference currently is an amount of local double uniformity. Entropy quantifies the grey level distribution unpredictability. Although the 14 features include information on the textural properties of the picture, it is difficult to determine the exact textural properties of each feature.

The wave energy characteristic represents the energy distribution across size and direction on the frequency axis and has been extremely effective for characterizing texture [31]. *x*(*m*, *n*) represents the subband numbers with the extreme histogram alteration of 1/*m*/*M* and also 1/*n*/*N*.

#### 3.1.3. Feature Selection

*Feature selection* is a method of selecting relevant characteristics to provide an effective and better solution to a particular issue. Ideally, an optimal subset of characteristics should be selected from the set of accessible features required and adequate for addressing the issue. Selection of features is essential since not all features offered are helpful. Some functions may be duplicated, while others during the learning period may create confusion. This increases the complexity of the function room needlessly, which in turn takes more computer time to learn or to find a solution to the provided issue.

*(1) Genetic Algorithms in Feature Selection and Weighting*. GAs are similar, iterative aspect optimizers that have successfully been used to a wide range of optimization issues, including numerous pattern recognition and classification applications. GA is inherently suited to selecting features since the issue has an exponential search area. Siedlecki and Sklansky's pioneering work (1989) showed that GA was superior to classical representative algorithms [32]. Much research was subsequently produced which showed the benefits of the GAs for feature selection [33–36]. As an optimization issue, the problem of dimensional decrease is healthy suitable for preparation.

**Figure 3:**
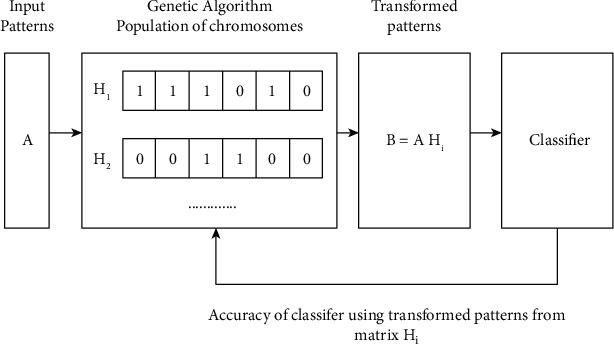
Feature extraction using genetic algorithm optimization with continuous wavelet transform.

GA aims at finding a transformed pattern in the d-dimensional input patterns in an *m* (*m* < *d*) dimension that optimizes the set of optimization criteria. In general, altered designs are assessed with their dimensionality and classification accuracy, rarely accomplished using a particular classifier in the patterns.  Figure 3 shows the design of an extractor based on the accuracy of GA classification as an assessment criterion. It holds a population with competitor matrices. Each matrix in this population is estimated by multiplying the input patterns and creating a sequence of changing patterns later, which are submitted to the classifier that segments the patterns into a training netlist for classifier learning and a test pattern for the accuracy of classification. To evaluate the quality of the transformation matrix used for collecting modified designs, the obtained precision is then returned to GA. The GA utilizes this information to seek a change that reduces the size of changing patterns and maximizes classification accuracy.

**Figure 4:**
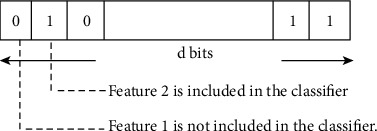
Single binary vectorization in genetic algorithm.

For Figure 4, the function selects each chromosome piece linked to a parameter and simplified to include *i*-th one bit as part of the modified classification patterns, or converse, if the bit is 0. According to their classification accuracy, every resultant characteristic group needs to be assessed on a set of test data [34]. The fitness value depends on the performance measurement of categorization, like categorization accuracy. Please note that in this instance of selection of features, the binary chromosomes do not contain actual value control factors: information about the existence or lack of features in the optimum classification set is incorporated in the bits themselves for avoiding the need for decoding.

Feature weighting determines the optimum collection of feature weights for the best chromosomes chosen via function selection techniques. In the suggested approach, each gene in the chromosome is given weight. Each weight is regarded as its associated categorization characteristic [37]. This implies that each characteristic is multiplied by its weight before categorization. The weights are limited to a specific interval. The weights of the essential characteristics tend to approach the maximum weight throughout the development. The opposite is true for the less significant characteristics, which behave more like noise and provide modest quantities of discriminatory categorization information. By multiplying features with optimum weights, the feature space is changed to optimize distances between distinct classes in the modified space. The optimum weights of the characteristics are somewhat necessary.

#### 3.1.4. Classification Using Adaptive Resonance Classifier Network

The initial neural network classification construction is ARCN, intended to cluster dual vectors via uncontrolled knowledge. This system controls the extent to identifying patterns that are similar inside one cluster [38]. The network may find clusters based on the data input. The network itself will store the information about clustering the patterns or features without prior knowledge of the potential cluster number and type. As soon as the first input pattern is produced, the network basically “follows the leader” to the next. The second group is created when the space of the first two clusters crosses a predetermined.

It creates a second cluster if the distance between the first and second clusters exceeds a precomputed threshold; otherwise, the pattern is categorized to the primary cluster, and a similar process is carried out for entire data sets.

**Figure 5:**
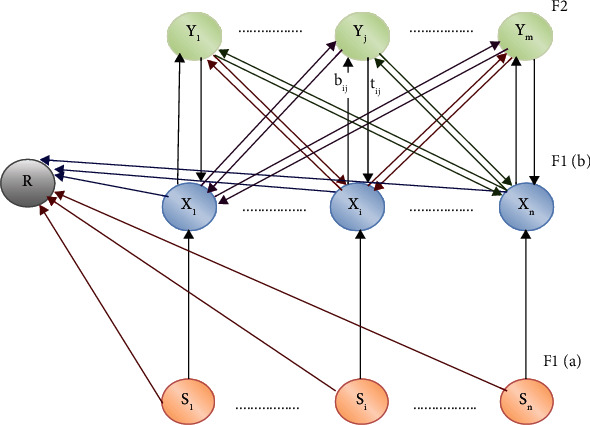
Overall architecture of proposed ARCN network.

*(1) ARCN Network Architecture*. As shown in Figure 5 of the ARCN net architecture [39], there are two cluster units and reset units used to check the degree of similarity between the patterns on the unit of a sole cluster. With the aid of two weighted route sets, the F1 layer is connected with F2.

Figure 5 represents the overall framework of a proposed classifier that consists of S input networks, *x* neurons, and *y* output layers. The classification process initiated and ends with the various neuron layers interconnected.

### 3.2. Comparison with Existing Algorithm

The computing unit architectures for ARCN comprise F1 components (participation and boundary components), F2 units (cluster units), and rearrange units that regulate users' patterns in a similar cluster degree. Each unit in layer F1(a) is linked to the appropriate unit in layer F1(b). Each unit in F1(a) and F1(b) has its reset unit linked to each unit of F2. Each unit in the F1(b) layer is linked by two weighted routes, each unit in the F2 (cluster) layer. A lower-up weight bij links the F1(b) unit Xi to the F2 unit Yj. Similarly, unit Yj is linked by top-down tji weights to unit Xi. F2 is the competitive layer with the nonzero activation of the uninhibited node with the most significant net input.

**Figure 6:**
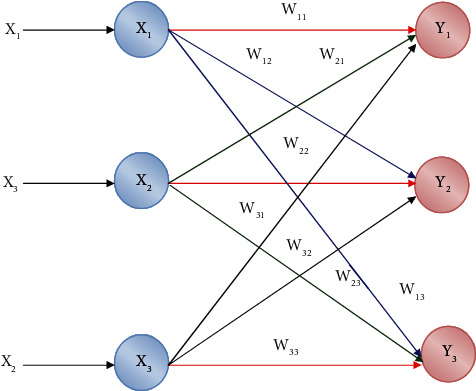
Classification network architecture existing in the literature.

The LVQ network architecture is shown in Figure 6 [40]. It is analogous to a Kohonen map creation with no topological framework to compare the construction of an LVQ network. Every output set is allocated to a specific set, which may be found here.

The existing networks and proposed networks have the major difference in the classification process. The weight and the network layer are different as compared to the existing layer as shown in Figure 6 and Table 3 represents the performance metrics of the proposed work that is calculated from True Positive and True Negative during the classification process.

Table 4 lists accuracy, sensitivity, specificity, F-measure, time, and classification. The average sensitivity for 500 samples is 91 percent, specificity is 98.4 percent, and classification is 92 percent. Precision using LVQ network is seen.

### 3.3. Resource Allocation with Proposed Algorithm

It is difficult to identify the many factors associated with NN, and determining the optimal configuration is time-consuming and requires patience. In BPNN, the MRAN method is employed to determine the smallest number of neurons needed to get included in the layer hidden to achieve maximum efficiency, rather than selecting the design at random or via trial and error. The multilayer perception (MLP) architecture is the most popular neural network architecture, and it is taught through backpropagation (BP) algorithms. The gradient descent technique has been proposed to decrease the mean network squared error as much as possible.

#### 3.3.1. Networks Present in ARCN

This model of MRAN incorporates the Resource Allocating Network (RAN) growth criteria of Platt with a pruning approach based on the relative contribution of each remote unit to the overall network output to create a sequential radial learning basis neural network that performs well in both training and testing. The resulting network results in a RAN design that is as simple as possible. Owing to the topological structure and openly revealing capability of the learning process, the RBFNN has been particularly compatible for pattern recognition and approximation of function [41, 42]. During the Radial Base Function (RBF) usage, the Gaussian functions were chosen from the essential functions, and the number of units (the Gaussian function widths and centers) can be determined depending on the input features. The output and remote unit weights are determined using a linear procedure described in detail. This method is not well adapted for sequential instruction is the only drawback and often results in an excessive number of hidden units. Platt contributed to addressing these constraints by creating an algorithm that, based on the new input, adds hidden units to the network. As a sequential learning technique, the approach is founded on the principle that the number of units contained in the data must be commensurate with the complexity of the underlying function. This is referred to as Resource Allocating Network (RAN), which begins with 0 hidden units and develops as more units of hidden are allocated, relying upon the novelty of consecutive interpretation. If an observation is made for the first time, the existing network parameters are adjusted using the LMS algorithm to fit the new observation.

#### 3.3.2. Layer 1 Resource Allocation

**Figure 7:**
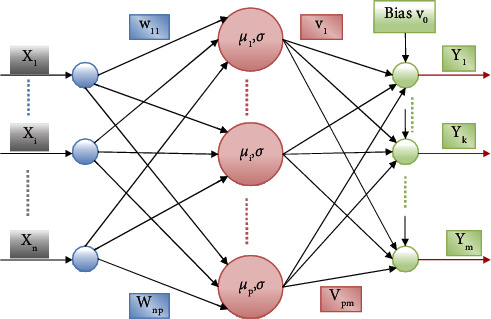
Architecture of proposed layers.

The RAN structure is similar to RBF [43], as depicted in Figure 7. There are two characteristics associated with each hidden unit inside the network, which are referred to as a center (*r*) and a width (*T*)

Radially symmetrical operation functions of the hidden units exist in the input space of the hidden units. Only the radial distance between the input vector and the corresponding hidden unit's center parameter will be dependent on the output of each hidden unit. Between the layer hidden and input, the weight ranges from w11 to wnp, and between the layer hidden and the output, the weight ranges from v11 to vpm. The response for each hidden unit is scaled to produce the total output network, which is accomplished via the connecting weights between the units hidden and the output.

Satisfying both aforementioned conditions, the data has been depicted as new, and a new unit hidden is placed into the data structure. Initially, the input is to be located at a distance from the other centers, and second, the error between the output of the network and the estimated output is significant in comparison to the goal output. The minimum precision needed for network output approximation is represented by the emin value, while the distance en reflects the input resolution scale represented by the distance en. The algorithm begins with the expression en = *e*_max_. Emax is chosen as the interest scale with the greatest range in the input space, which is often the whole nonzero probability input field. The distance has decreased as en = max*e*_max_, *n*, *e*_min_, where 0 indicates a steady decay and 1 represents a rapid decay. The value of en is decreased to the value of *e*_min_. Based on a growing number of observations, the distance criterion may be fine-tuned by using fewer base functions with broad widths (smoother base functions) initially, and lesser width basic functions are assigned to correct the approximation as the number of observations increases. “k” denotes the overlapping factor defining the overlay in the hidden unit input space replies. The value of emax and emin is 0.4 and 0.2.

**Figure 8:**
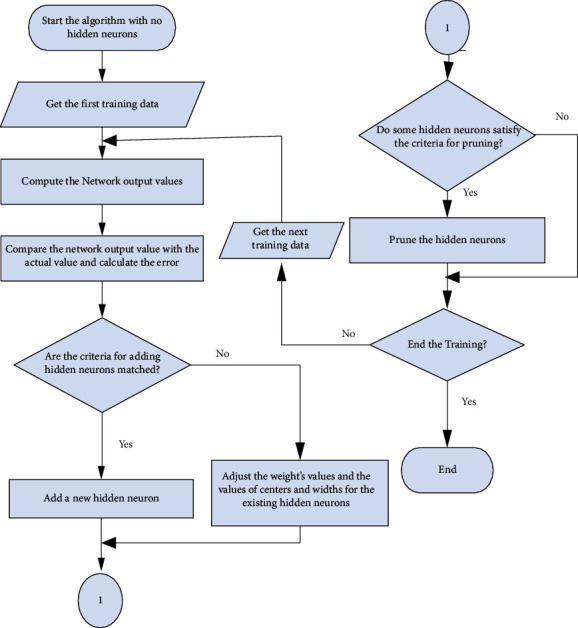
MRAN algorithm flowchart.

Figure 8 shows the flowchart of the MRAN algorithm. It provides details on the calculation of output values of the network through comparison with the real values to get the error value. If the error meets the additional requirement of newly created neurons, then the existing neurons' weight, center, and breadth are modified to meet the conditions using the new hidden neuron. When the requirements for tailing are met by the neurons hidden, for tailoring the hidden neurons, the training takes place if it does not finish.

*(1) Minimal Resource Allocation Network (MRAN) Training*. The MRAN network is trained by the EGG database. The target is given to 1 for the right position and 0 for the incorrect categorization position. The minimal number of hidden layer neurons (HLN) needed for optimal efficiency is determined using the MRAN method.

**Figure 9:**
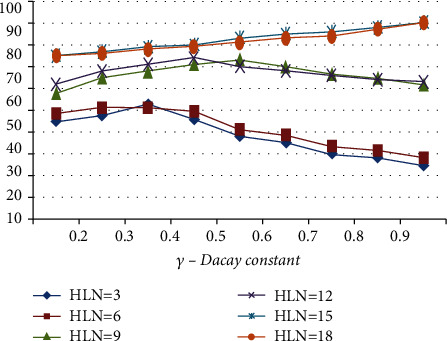
MRAN algorithm-based classification for differing decay constant.

Using various decay constant values related to hidden neurons in the layer, Figure 9 depicts the MRAN network consistency in the classification of illness for various decay constants values in the layer [44]. When training MRAN with values lesser compared to 9 HLN, it has been observed that the percentage of classification is lower than expected. For a decay constant of 0.9, 90 percent of the classification is saturated at the 15-point level and above. It has also been noticed that the classification increases linearly with a distinct decreasing constant value for each of the 15 HLN numbers tested. The number of HLN used ranges from 9 to 18 to configure HLN in the BPNN architecture to obtain the highest possible categorization percentage.

Table 5 lists the various research in which MRAN has provided unique neurons, as well as the effects associated with those neurons. The MRAN technique is employed to determine the number of neurons that are required in the layer hidden to achieve maximum efficiency. In the network, 350 samples are being analyzed. Table 4 shows that the 15-neuron network performs at its highest possible efficiency level. Furthermore, the amount of correctly classified [45] for each disease is shown.

Thus, the network [46] set-up using the MRAN algorithm is fixed at 60-15-7. This design is then utilized to classify digestive diseases. Classification is applied by several training algorithms such as traingdx, trainrp, trainross, trainlm, trainscg, traincgp, trainbfg, traincgf, and traincgb. Using accurate classification, the comparison of the training algorithm has been performed.

**Figure 10:**
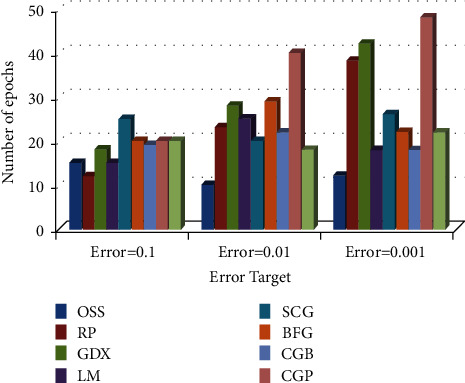
Comparison chart of the number of epochs for normal subjects concerning error targets.

**Figure 11:**
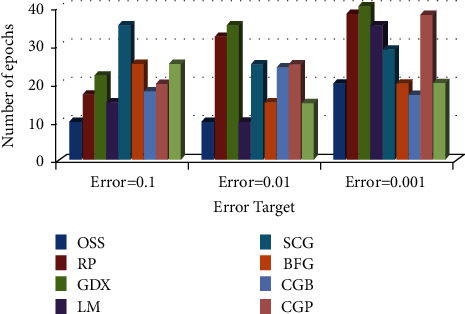
Comparison chart of the number of epochs for *bradygastria* subjects concerning error target.

**Figure 12:**
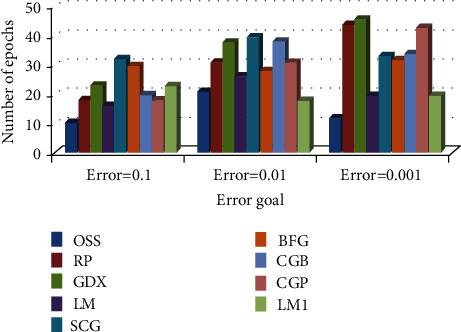
Comparison chart of the number of epochs for dyspepsia subjects concerning error target.

**Figure 13:**
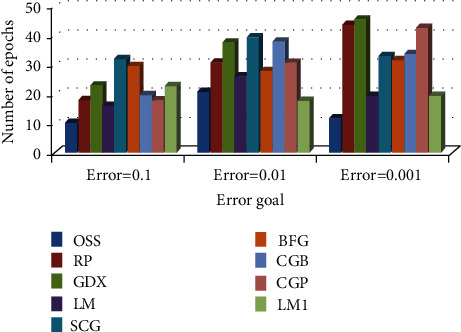
Comparison chart of the number of epochs for nausea subjects concerning error target.

**Figure 14:**
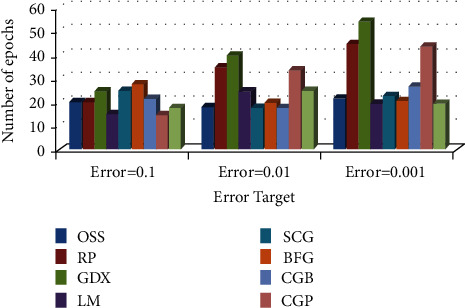
Comparison chart of the number of epochs for tachygastria subjects concerning error target.

**Figure 15:**
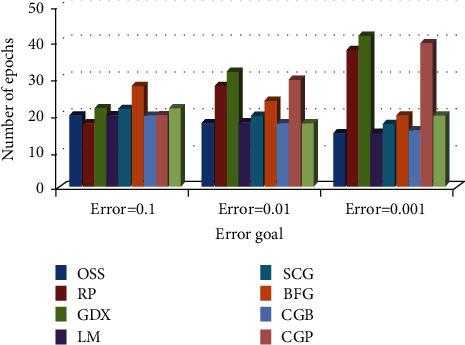
Comparison chart of the number of epochs for ulcer subjects concerning error target.

**Figure 16:**
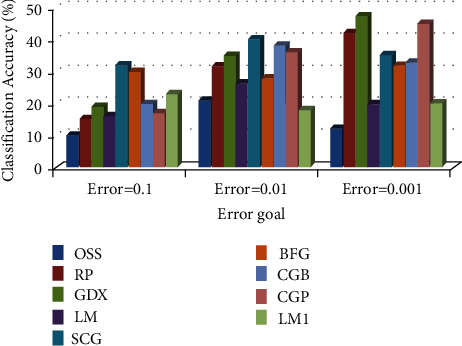
Comparison chart of the number of epochs for vomiting subjects concerning error target.

*(2) Comparison of Training Algorithm Performance in BP-MRAN Network*. Figures 10–16 are presented in the performance comparisons of various training algorithms for normal individuals, subjects of *bradygastria*, subjects of dyspepsia, subjects of nausea, subjects of tachygastria and of ulcers, and subjects of vomiting. The diagram is drawn between the error objective and the periods. From all graphs, the epoch values of trainrp, traingdx, and traincgp have been increasing for different error target values [47].

Figure 10 presents the performance comparisons of various training algorithms for normal individuals and subjects of *bradygastria*.

Figures 11 presents the performance comparisons of various training algorithms for normal individuals.

The categorization of EGG topics using this BP-MRAN architecture takes place with a variable rate of learning and the value of momentum factor for various algorithms of training. Table 5 shows the highest efficiency of every method for every rate of learning and dynamic factor.

The categorization of EGG patients [48] with trainrp is seen in Table 6 at 97% for LR and MF 0.6. Compared to other training algorithms, the trainrp has superior performance. Also, it has been observed that a maximum classification of 61% was recorded with 0.2 Lr and 0.8 M for Trainoss. A maximum classification of 94% for Lr and 0.4 M was obtained from traingdx algorithm, whereas trainlm algorithm yields a maximum 56% classification for the Lr value 0.4 and 0.6. Meanwhile, the trainscg algorithm yields a maximum 65% classification with 0.4 Lr and 0.8 M. The trainbfg algorithm was rated at a maximum level of 63% with 0.6 Lr and 0.8 M, the traincgb training algorithm attained a maximum 62% rating for 0.6 Lr and *M*, the traincgp training algorithm [46] was rated at a maximum 93% level, and the traencgf training algorithm was rated at 60% maximum level.

MRAN, which is employed with the combination of BPNN, determines the total amount of HLNs for obtaining optimum classification efficiency [49]. Considering the data of 500 samples, results are calculated following the prior comparative confusion research matrix [50].

## 4. Confusion Matrix for BP-MRAN Network

For various sample sets, a confusion matrix [51] for signals collected in the laboratory setting of varied compositions was used.

## 5. Neural Comparison Architectures

This research work will describe the results for the three designs utilized to identify anomalies in the EGG signals. The specificity and accuracy of classification for three different designs are compared [52]. The sensitivity and specificities indicate that all architectures are described in Figures 15 and 16. As demonstrated in Figure 16, the classification precision for the three designs is compared. The result shows that a 69.5% accuracy of classification [53] was obtained for ARCNN design, 92.0% was obtained for LVQNN, and 96% was obtained for BP-MRAN.

## 6. Results and Discussion

In this work, three artificial neural network (ANN) architectures have been built and tested to categorize EGG signals. The EGG is classified as normal or abnormal using the ARCNNN network, an unsupervised network. The LVQ network is investigated as a supervised method that employs competing layers to improve the accuracy of the classification decision-making process. The BPNN was implemented via the use of supervised learning. Using the MRAN technique, optimizing the architecture's efficiency while simultaneously reducing computation time is possible. The performance of the BP-MRAN training algorithm is compared to that of nine other training algorithms. The most significant percentage of trainrp, traingdx, and traincgp that can be classified is 96.28 percent, 94 percent, and 92.57 percent, respectively. To compare the sensitivity of different methods, an analysis of specification is performed, and 96% accuracy of classification occurred using BP-MRAN with the combination of a robust algorithm of backpropagation. It was found that the BP-MRAN with trainrp improved performance by 14 percent and 10 percent, respectively, in comparison to the results obtained by Chacon et al. using the BPANN in a combination of trainrp [54] and the results obtained by Curilem et al. using the GA and SVM [55].

## 7. Conclusion

Stomach disorders bring significant changes in the physical health system, which also affects the mental health of humans. The proposed work uses various machine learning algorithms to classify stomach disorders. The primary goal was to categorize electrogastrography data into two or three classes accurately. The electrogastrography signals are classified into three classes (normogastia, bradygastria, and tachygastria) and two classes. In future work, various deep learning algorithms can be carried out with the proposed technique, which would give better results while using many datasets.

## Figures and Tables

**Table 1:** Comparison of Existing works.

Authors	Stomach position	Electrode position	Coordinates	Disadvantage
[19]	Cutaneous reference points	Proximal electrodes	Placed in the costal margin of the stomach	Commonly used but time consumption
[20]	Sonography method	2 electrodes	Left side of the abdomen and ventral region	Overuse of electrodes may lead to severe problems in accuracy rate
[21]	Cutaneous reference points method	1 electrode	Between umbilicus and xiphoid process	Low signal to noise ratio
[22]	X-ray method	6 electrodes	At least in one channel of the abdomen	Low signal to noise ratio
[23]	Cutaneous reference points method	5 electrodes	Umbilicus and xyphoid process	Pick up propagation is too low

**Table 2:** Categorization of dataset.

EGG	Mean Age (years)	Male	Female
Normal	33	32	18
Bradygastria	28	27	23
Dyspepsia	38	29	21
Nausea	45	28	22
Tachygastria	36	26	24
Ulcer	34	31	19
Vomiting	35	19	31

**Table 3:** Feature Extraction of proposed work.

Feature number	Input features
1, 2	Second rotational momentum
3, 4	Dissimilarity
5, 6	Correlation \variance
7, 8	Moment of reverse difference
9, 10	The average sum
11, 12	Their variance
13, 14	Entropy sum
15, 16	Entropy
17, 18	Variance of difference
19, 20	Entropy difference
21, 22	Correlation information measure I
23, 24	Correlation information measure II
25, 26	The maximum coefficient of correlation
27, 28	Second angular moment

**Table 4:** Analysis of performance metrics with proposed technique.

S. No.	Input signals	Proposed technique					
		Precision	Sensitivity	Specificity	Input measure	Frequency (sec)	Overall accuracy (%)
1	268	0.82	0.864	0.956	0.874	14	91.2
2	389	0.89	0.893	0.978	0.896	19	92.4
3	457	0.90	0.912	0.956	0.945	25	93.9
4	562	0.91	0.923	0.936	0.969	21	97.6

**Table 5:** Proposed algorithm parameters.

Trials	No. of neurons in the hidden layer	Amount of test EGG classified (50 each)							Total	Correct classification (%)
		Normal	Abnormality							
			Dyspepsia	Tachygastria	Nausea	Vomiting	Ulcer	Bradygastria		
1^st^	9	44	39	43	46	34	27	48	372	77.43
2^nd^	22	43	37	43	39	48	39	49	398	86.24
3^rd^	23	36	33	33	30	43	47	43	333	63.72
4^th^	24	46	40	39	43	48	46	44	303	86.67
**5** ^**th**^	**26**	**46**	**42**	**48**	**44**	**49**	**38**	**60**	**326**	**95.00**
6^th^	26	43	40	43	46	60	44	60	326	90.00
7^th^	27	43	43	44	46	48	44	49	326	90.00

**Table 6:** EGG classification accuracy for various training algorithms with several values of *α* and *β*.

Lr † (*α*)	M ∓ (*β*)	EGG correct classification (%)								
		OSS	RP	GDX	LM^*∗*^	SCG	BFG^*∗*^	CGB	CGP	CGF
0.2	0.2	48	81	82	50	54	50	47	82	44
	0.4	52	83	84	52	56	52	52	84	46
	0.6	58	84	88	51	60	58	58	88	48
	0.8	**61**	86	90	53	58	61	59	90	51

0.4	0.2	45	85	92	52	61	48	45	92	45
	0.4	48	87	**94**	54	63	48	48	94	48
	0.6	52	91	93	**56**	62	52	52	93	52
	0.8	58	95	92	55	**65**	60	58	92	58

0.6	0.2	50	93	91	53	61	52	54	91	50
	0.4	52	95	93	51	63	58	58	93	52
	0.6	58	**97**	92	54	62	60	**62**	92	58
	0.8	60	96	90	52	64	**63**	61	90	**60**

0.8	0.2	52	90	90	52	60	52	52	90	52
	0.4	54	91	93	53	62	54	54	**93**	54
	0.6	59	92	92	55	63	56	59	92	59
	0.8	60	95	91	54	62	58	60	91	57

†Lr: rate of learning, ∓ M: momentum factor, ^*∗*^-M is not applicable.

## Data Availability

The data that support the findings of this study are available on request from the corresponding author.
